# TRPV4 Increases the Expression of Tight Junction Protein-Encoding Genes via XBP1 in Mammary Epithelial Cells

**DOI:** 10.3390/ani10071174

**Published:** 2020-07-10

**Authors:** Md Aminul Islam, Moeko Mizusawa, Mst Mamuna Sharmin, Satoko Hayashi, Shinichi Yonekura

**Affiliations:** 1 Department of Biomedical Engineering, Graduate School of Medicine, Science and Technology, Shinshu University, Minamiminowa, Kamiina 399-4598, Nagano, Japan; 18hb151d@shinshu-u.ac.jp (M.A.I.); 18hb152b@shinshu-u.ac.jp (M.M.S.); 20hb103c@shinshu-u.ac.jp (S.H.); 2 Department of Biomedical Engineering, Graduate School of Science and Technology, Shinshu University, Minamiminowa, Kamiina 399-4598, Nagano, Japan; 18bs116a@shinshu-u.ac.jp; 3 Department of Biomolecular Innovation, Institute for Biomedical Sciences, Shinshu University, Minamiminowa, Kamiina 399-4598, Nagano, Japan

**Keywords:** HC11 mouse mammary epithelial cell, TRPV4, UPR, mammary gland development

## Abstract

**Simple Summary:**

Mammary glands are exocrine tissue, capable of secreting adequate amounts of milk protein during lactation. Each mammary gland is occupied by numerous alveoli. Each alveolus is composed of a single layer of mammary epithelial cells, adipose tissue, and ducts. Recent studies indicate that mild heat treatment of mammary epithelial cells at 39 °C has activated milk production. These results suggest that temperature may influence the physiological functions of mammary epithelial cells. In this study, we found that the temperature-sensitive transient receptor potential vanilloid 4 (TRPV4) was involved in the increase of *β-casein* and TJ protein-encoding gene expression in response to mild heat treatment. On the other hand, severe heat treatment (41 °C) reduced the cell viability. Moreover, the *Trpv4* mRNA level was significantly increased at Day 15 of gestation when the mammary alveoli are formed. TRPV4 is activated not only by temperature but also by mechanical forces that guide mammary epithelial development in the normal mammary gland. Our data suggest that TRPV4 has a possible function in mammary gland development.

**Abstract:**

Mild heat stress (39 °C–40 °C) can positively regulate cell proliferation and differentiation. Indeed, mild heat treatment at 39 °C enhances the less-permeable tight junctions (TJs) formation and milk production in mammary epithelial cells. However, the molecular mechanisms of this response have not yet been delineated. In this study, the involvement of temperature-sensitive transient receptor potential vanilloid 4 (TRPV4) in the increase of *β-casein* and TJ protein-encoding gene expression in response to mild heat treatment (39 °C) has been explored using HCll mouse mammary epithelial cells. Severe heat treatment (41 °C) induced the transcriptional level of *Chop* (C/EBP homologous protein; proapoptotic marker) and reduced the cell viability. It is speculated that the difference in unfolded protein response (UPR) gene expression upon stimulation at 39 °C vs. 41 °C controls cell survival vs. cell death. The accumulation of *Trpv4* mRNA was significantly higher in 39 °C heat treatment cells. The *β-casein*, *Zo-1* (zona occludens-1), *Ocln* (occludin), and *Cldn3* (claudin 3) transcript levels were significantly increased in response to the addition of a selective TRPV4 channel agonist (GSK1016790A) at 37 °C. TRPV4 stimulation with GSK1016790A also increased the X-box-binding protein 1 splicing form (*Xbp1s*) at the transcript level. The increase in the mRNA levels of *β-casein*, *Zo-1*, *Ocln*, and *Cldn3* in response to 39 °C heat treatment was suppressed by XBP1 knockdown. Moreover, the transcript level of *Trpv4* was significantly increased at Day 15 of gestation, and its expression declined after 1 day of lactation. TRPV4 is activated not only by temperature but also by mechanical forces, such as cell stretching and shear stress, which guide mammary epithelial development in a normal mammary gland. These findings provide new insights of the possible function of TRPV4 in mammary gland development.

## 1. Introduction

Mammary glands are exocrine tissue, capable of secreting adequate amounts of milk protein during lactation. Each mammary gland is occupied by numerous alveoli. Each alveolus is composed of a single layer of mammary epithelial cells, adipose tissue, and ducts. During mid-gestation, mammary alveoli are made, and the development of the mammary gland ends by late gestation [1]. The polarization of mammary epithelial cells has occurred with apical (luminal) and basolateral surfaces [2]. Tight junctions (TJs) are formed on the apical surface of the neighboring cells whereby particularity in the entrance of the solutes between the lumen and the basolateral bloodstream is established [3]. Moreover, the less-permeable TJs form immediately after parturition and it is maintained throughout lactation [4].

Hormesis is a phenomenon that induces adaptive responses in cells when exposed to various stressors, including mild heat stress, etc. [5]. Mild heat stress (39 °C–40 °C) can positively regulate cell proliferation and differentiation [6]. Indeed, a previous study has shown that heat-shock at 39 °C has activated milk production and enhanced the formation of less-permeable TJs [7,8]. However, detailed molecular mechanisms of the mammary epithelial cell response to mild heat shock have not yet been elucidated.

Transient receptor potential (TRP) receptors on cellular membranes are sensitive to heat stimulation, nine of which have been identified so far [9]. TRP receptors are expressed in many tissues, such as the mammary glands [10,11]. TRP may be liable for Ca^2+^ transport in mammary gland epithelial cells. However, there is scant information regarding TRP expression in mammary epithelial cells. The localization of transient receptor potential vanilloid 4 (TRPV4) occurs in the basolateral membrane compartment of mouse mammary epithelial cells and acts as a calcium-permeable channel [12]. Moreover, TRPV4 can also be activated by warm temperatures (>34 °C) and mechanical forces, such as cell stretching and shear stress [13]. It is possible that TRPV4 is involved in intracellular signaling, which is activated by temperature changes in mammary epithelial cells.

Heat stress induces endoplasmic reticulum stress, which elicits an unfolded protein response (UPR) [14,15]. The UPR is initiated via stimulating three arms: activating transcription factor 6 (ATF6), protein kinase R-like ER kinase (PERK), and inositol-requiring enzyme 1 alpha (IRE1α) [16,17]. Activated IRE1α enhances the unconventional splicing of *Xbp1* (X-box binding protein 1) mRNA (*Xbp1u*). Thereby *XBP1u* leaves an intron of 26 bp and generates the XBP1 splicing form (X*bp1s*) as the transcription factor [18]. We have previously reported that the UPR-related transcription factor XBP1 is involved in the differentiation of HC11 mouse mammary epithelial cells [19]. Moreover, we recently revealed that XBP1 plays a role in regulating the expression of *β-casein* following 39 °C heat shock [8]. β-casein is a differentiation marker in HC11 cells and a representative milk protein.

In this work, the aim was to determine whether both TRPV4 and XBP1 are involved in the increase of the TJ protein-encoding gene and *β-casein* expression upon mild heat stress. Moreover, we investigated the mRNA expression level of *Trpv4* at different stages of mammary gland development.

## 2. Materials and Methods

### 2.1. Reagents

Fetal bovine serum (FBS) was obtained from EQUITECH-BIO (Kerrville, TX, USA). Dulbecco’s modified Eagle medium (DMEM, 4500 mg/L) was purchased from SIGMA-ALDRICH (Cat#D7777; St. Louis, MO, USA). Epidermal growth factor, penicillin/streptomycin, TRPV4 agonist (GSK1016790A), and anti-alpha smooth muscle actin (α-SMA) were purchased from SIGMA-ALDRICH. Goat anti-mouse IgG Alexa Fluor^®^ 488 and goat anti-mouse IgG Alexa Fluor^®^ 568 were purchased from Life Technologies (Carlsbad, CA, USA). Anti-TRPV4 was bought from Alomone labs (Jerusalem BioPark, JBP, Israel). Other chemicals were purchased from Nacalai Tesque (Kyoto, Japan).

### 2.2. Animal

BALB/c wild-type mice were obtained from Japan SLC (Shizuoka, Japan). The mice were provided with a standard pellet diet and water ad libitum. They were maintained in a temperature-controlled environment with a 12 h light–dark cycle. Non-pregnant mice aged 8–13 weeks were mated to generate pregnant mice. Day 1 of pregnancy was confirmed by observing the presence of a copulatory plug. Mammary glands were isolated at gestation (15 days) and lactation 1 and 7 days from non-pregnant and pregnant mice. Then the mammary gland tissue samples were collected. All experiments were performed according to guidelines approved by Shinshu University for the welfare of laboratory animals.

### 2.3. Cell Culture and Transfection

Cell culture and transfection were performed as previously described [8]. HC11 cells (a gift from Dr. Takaharu Kozakai, Yamagata University, Yamagata, Japan) were cultured in DMEM containing 1% penicillin and streptomycin and 10% FBS with 10 ng/mL epidermal growth factor. Cultures were maintained at 37 °C in a 5% CO_2_ incubator. HC11 cells were grown to 100% confluence and subjected to heat treatment or treated with 10 μM GSK1016790A.

In case of transient infection, Lipofectamine 2000 (Life Technologies) was used to transfect the cells as per the manufacturer’s instructions. The used siRNA (Xbp1 small interfering RNA) and control siRNA were taken from Sigma-Aldrich and Santa Cruz (Santa Cruz, CA, USA), respectively.

### 2.4. RNA Extraction and RT-qPCR

Total RNA was extracted from the HC11 cells using TRIzol reagent (Life Technologies) following the manufacturer’s protocol. A qPCR RT Master Mix with gDNA Remover (Toyobo, Osaka, Japan) was utilized for reverse transcription. Quantitative real-time PCR was carried out using the SYBR Premix Ex TaqTM ll (TaKaRa Biotechnology, Kusatsu, Japan), to quantify the relative expression level of mRNA. Relative mRNA expression was estimated by the double delta delta Ct method and represented as the relative values to the control. GAPDH was used as the housekeeping gene. The primers used for the quantitative PCR are shown in Table 1. The reaction sensitivity and amplification of the contaminating products were examined by amplifying serial dilutions of cDNA.

### 2.5. Immunohistochemistry

BALB/c mouse mammary gland tissues were collected from 10-week-old virgin animals and fixed in 4% paraformaldehyde for 48 h. Paraffin sections with 4 μm slices were prepared. Then the sections were kept at 60 °C for 15 min. After incubation the paraffin was removed using xylene and again hydrated in a graded ethanol series. Antigen retrieval was performed using Immunosavor (Nisshin EM Co., Ltd., Tokyo, Japan). A 10% Goat Serum (Life Technologies) was used for blocking the nonspecific binding sites of the deparaffinized sections. Then the sections were kept overnight at 4 °C with anti-TRPV4 or anti-α-SMA. Afterwards, the sections were washed with PBS and incubated with Alexa Fluor^®^ 488 goat anti-rabbit IgG and Alexa Fluor^®^ 568 goat anti-mouse IgG for 2 h. EVOS^®^ FL Auto (Life Technologies) was used to capture the fluorescence photographs.

### 2.6. Cell Viability Test

HC11 cells were cultured at a density of 2 × 10^3^ cells/well on 96-well plates. After 48 h, cells were placed in an incubator at 39 °C or 41 °C for 72 h or 6 h, respectively. Cell viability was measured using the MTT Cell Viability Assay Kit (Biotium, Fremont, CA, USA) according to the manufacturer’s protocol. Briefly, 10 μL of the MTT solution was added to 100 μL of culture medium. After 4 h of incubation at 37 °C, 200 μL of dimethylsulfoxide was added to each well. The absorbance was measured using a multimode microplate reader (iMark microplate reader, Bio-Rad, Hercules, CA, USA) at 570 nm and a reference wavelength of 630 nm.

### 2.7. Statistical Analysis

Data are presented as the mean values ± standard error of the mean from at least 3 replicates in each experimental group. Data were analyzed to calculate the statistical difference between the control and treatment group using a Tukey–Kramer test or Student’s *t*-test. A *p*-value less than 0.05 was considered significant.

## 3. Results

### 3.1. Effects of Different Temperatures on mRNA Levels of UPR-, TJ Protein-Related Genes, and Cell Viability

**Figure 1:**
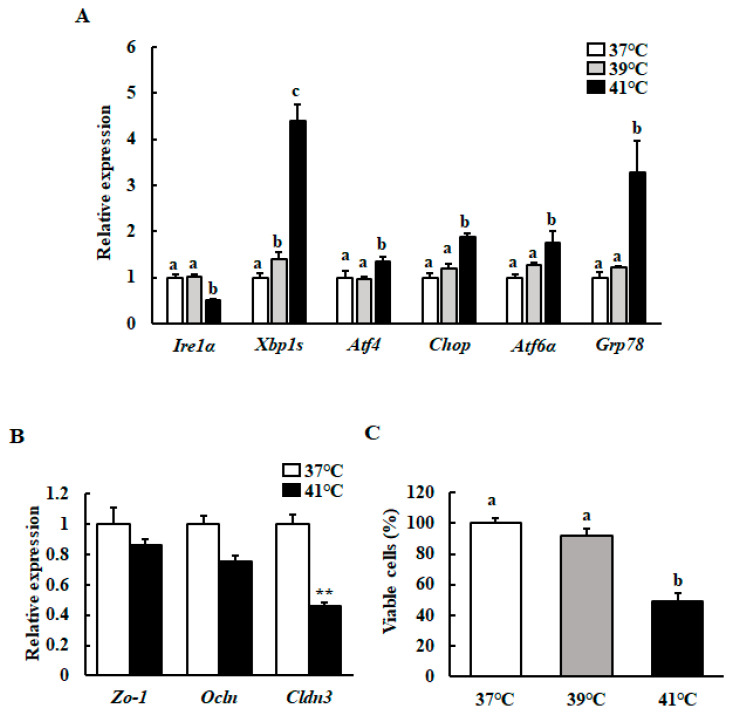
Effect of different temperatures on the mRNA levels of the UPR- and TJ protein-related genes, as well as on cell viability. After reaching 90%–100% confluence, HC11 cells were incubated at 37 °C (control), 39 °C for 72 h, or 41 °C for 6 h. (**A**) The mRNA level of *Ire1α*, *Xbp1s*, *Atf4*, *Chop*, *Atf6α*, and *Grp78* were determined using RT-qPCR and normalized to that of GAPDH. (**B**) The mRNA level of *Zo-1*, *Ocln*, and *Cldn3* were determined using RT-qPCR and normalized to that of GAPDH. (**C**) Cell viability was measured using an MTT assay. The results are expressed as the mean values ± SEM for four independent experiments. Means with different letters are significantly different, *p* < 0.05. ** *p* < 0.01 compared to the control.

We used qRT-PCR to investigate the mRNA levels of the UPR- and TJ protein-related genes in HC11 cells cultured at 39 °C or 41 °C. We revealed that the transcript levels of *Atf4* (activating transcription factor 4), *Chop* (C/EBP homologous protein), *Atf6α*, and *Grp78* (glucose-regulated protein 78) were significantly increased and *Ire1α* expression decreased with heat treatment, but only at 41 °C. However, the expression level of *Xbp1s* was significantly increased with both mild and severe heat treatment (Figure 1A). Heat treatment at 41 °C significantly downregulated the *Cldn3* mRNA levels compared with the control (Figure 1B). We further examined whether heat treatment affected HC11 cell viability using an MTT assay. Only heat treatment at 41 °C significantly decreased cell viability (Figure 1C).

### 3.2. Mild Heat Treatment Increases Trpv4 mRNA Levels in HC11 Cells

**Figure 2:**
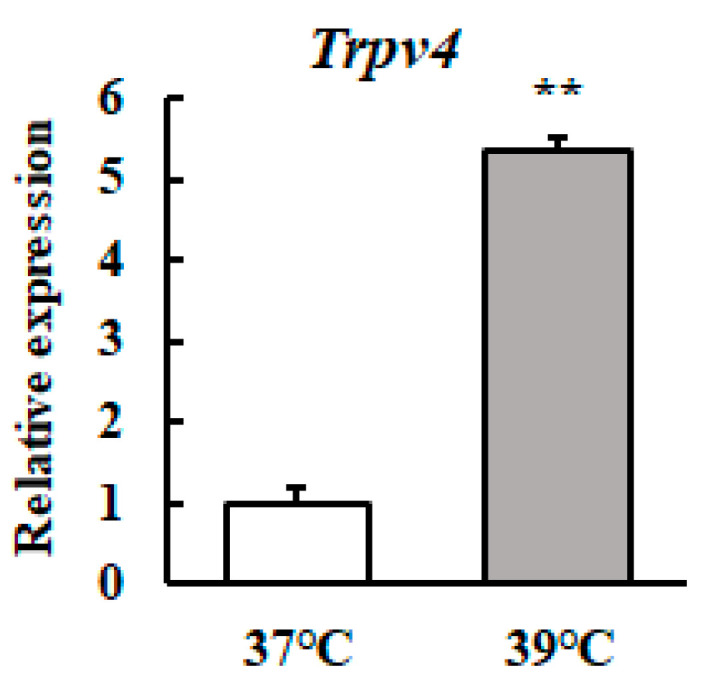
Heat treatment at 39 °C increases the *Trpv4* mRNA level in HC11 cells. HC11 cells were maintained at 37 °C or exposed to 39 °C for 72 h. The *Trpv4* mRNA level was detected by RT-qPCR. GAPDH was used as the reference gene. The results are expressed as the mean values ± standard error of the mean from four independent experiments. ** *p* < 0.01 compared to the control.

We investigated whether the *Trpv4* transcript level in mammary epithelial cells was affected by mild heat treatment. The *Trpv4* mRNA level was significantly higher in mild heat treatment cells (Figure 2).

### 3.3. TRPV4 agonist Increases β-casein and TJ Protein-Encoding mRNA Levels

**Figure 3:**
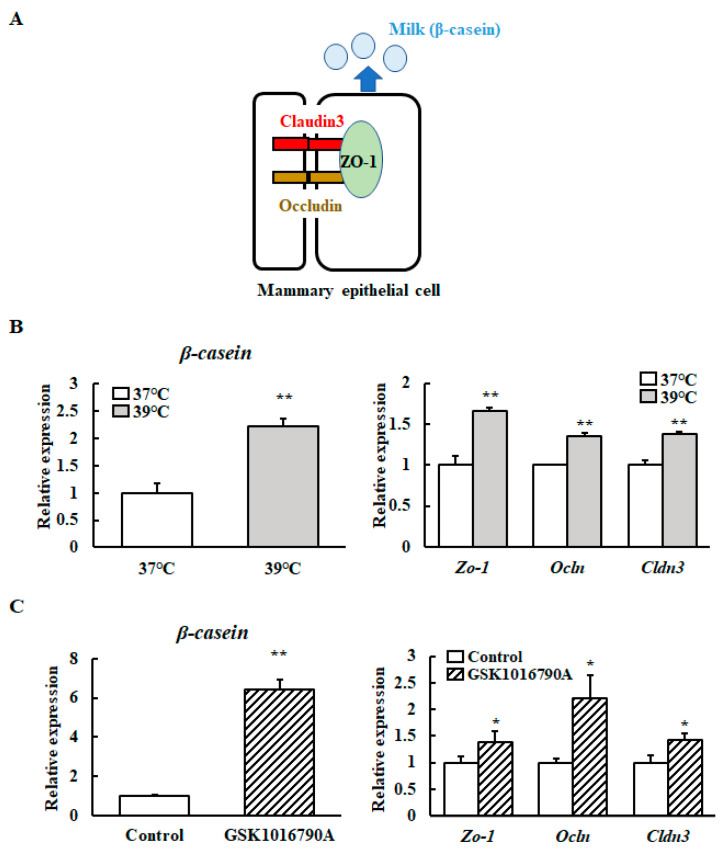
Levels of *β-casein* and TJ protein-encoding gene transcripts with and without a TRPV4 agonist. (**A**) Schematic diagram of Cloudin3, Occludin, ZO-1, and β-casein in mammary epithelial cells. (**B**) HC11 cells were maintained at 37 °C or exposed to 39 °C without differentiation medium for 72 h or (**C**) HC11 cells were incubated at 37 °C without (control) or with 10 μM GSK1016790A for 24 h. The mRNA levels of *β-casein*, *Zo-1*, *Ocln*, and *Cldn3* were detected by RT-qPCR. GAPDH was used as the reference gene. The results are expressed as the mean values ± standard error of the mean from four independent experiments. * *p* < 0.05 and ** *p* < 0.01 compared to the control.

We investigated the effect of mild heat treatment on the TJ protein-encoding gene and *β-casein* transcript levels in mammary epithelial cells using RT-qPCR. The *Zo-1* (Zona Occludens-1), *Ocln* (Occludin), *Cldn3* (Claudin 3), and *β-casein* mRNA levels were significantly higher in heat-shocked mammary epithelial cells (Figure 3A). We further investigated the *Zo-1*, *Ocln*, *Cldn3*, and *β-casein* transcript levels in mammary epithelial cells cultured with a selective *TRPV4* channel agonist (GSK1016790A) at 37 °C. The mRNA levels of *Zo-1*, *Ocln*, *Cldn3*, and *β-casein* were significantly increased in response to the addition of GSK1016790A (Figure 3B).

### 3.4. Mild Heat Shock at 39 °C Increases β-casein and TJ Protein-Encoding Gene Transcript Levels via XBP1

**Figure 4:**
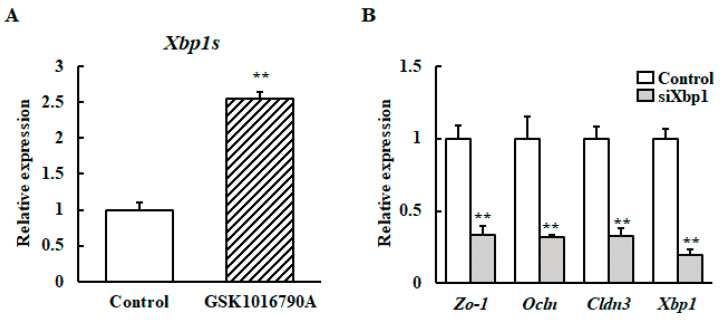
Effect of XBP1 knockdown on the mRNA expression of *β-casein* and the TJ protein-encoding gene after heat stimulation at 39 °C. (**A**) HC11 cells were incubated at 37 °C without (control) or with 10 μM GSK1016790A for 24 h. The mRNA level of *Xbp1s* was detected by RT-qPCR. GAPDH was used as the reference gene. (**B**) HC11 cells were transfected with XBP1 siRNA or control scramble siRNA for 24 h. Then the cells were maintained at 37 °C or exposed to 39 °C without differentiation medium for 72 h. The mRNA level of *Zo-1*, *Ocln* and *Cldn3* were detected by RT-qPCR. GAPDH was used as the reference gene. The results are expressed as the mean values ± SEM for four independent experiments. ** *p* < 0.01 compared to the control.

The transcript levels of *Xbp1s* in the HC11 cells cultured with GSK1016790A at 37 °C were investigated to determine whether TRPV4 activity can induce *Xbp1s* transcript accumulation. TRPV4 activation with GSK1016790A increased the *Xbp1s* transcript level (Figure 4A).

Next, it was examined if XBP1 is involved in upregulation of the TJ protein-encoding gene or *β-casein* mRNA upon mild heat treatment. We generated XBP1 knockdown of HC11 cells using *Xbp1* small interfering RNA (siRNA). After mild heat treatment, the *Zo-1*, *Ocln*, *Cldn3*, and *β-casein* mRNA levels were analyzed using RT-qPCR. Results reveal that the increased mRNA expressions of *β-casein*, *Zo-1*, *Ocln*, and *Cldn3* by mild heat treatment was inhibited by Xbp1 silencing (Figure 4B).

### 3.5. Change in Trpv4 Expression During Mammary Gland Development

**Figure 5:**
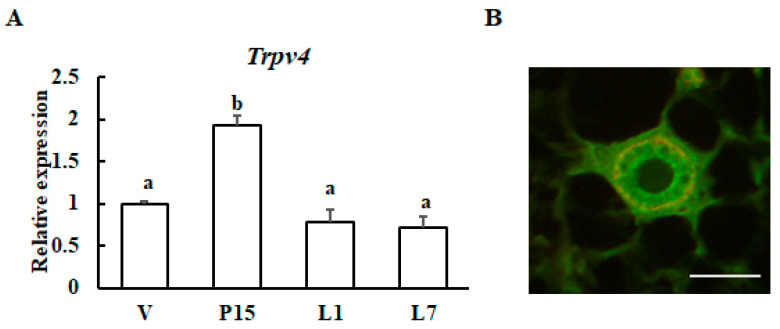
Expression patterns of the *Trpv4* gene during mammary gland maturation. (**A**) RT-qPCR analysis of *Trpv4* mRNA in the mouse mammary glands during pregnancy and lactation: virgin (V); 15-day pregnant (P15); 1-day lactation (L1); and 7-day lactation (L7). The results are expressed as the mean values ± SEM for four individual mice. Different letters represent significant difference, *p* < 0.05. (**B**) Immunohistochemical staining of TRPV4 (green) and smooth muscle actin (red) in mammary glands from 10-week-old virgin animals. Scale bar = 50 μm.

Next, the expression levels of *Trpv4* were examined at different stages of mammary gland development by qRT-PCR analysis. The transcript level of *Trpv4* was significantly increased at Day 15 of gestation, and its expression declined after 1 day of lactation (Figure 5A). Secondly, TRPV4 protein localization was examined in mammary gland tissue by immunohistochemical staining. We confirmed that TRPV4 was located in mammary epithelial cells (Figure 5B).

## 4. Discussion

Our study indicates that the transcript level of *Trpv4* was significantly higher in cells cultured at 39 °C than that in cells cultured at 37 °C. Previous studies suggested that, under many different experimental conditions, TRPV4 maintains a substantial degree of temperature-dependent activity over a range of temperatures (between 30 °C and 40 °C) [20,21]. Therefore, mild heat treatment induces TRPV4 activity in mammary epithelial cells.

Previous studies indicated that the activation of TRPV4 strengthens the TJ-associated barrier through the upregulation of *Ocln* in skin keratinocytes and corneal epithelial cells [22,23]. Conversely, the TRPV4 activator (4α-PDD) decreased the expression level of Cldn3 in HC11 cells [12], which is different from our results. It is reported that GSK1016790A is a potent and selective agonist of TRPV4 in different cell types [24] and that 4α-PDD is 300-fold less potent than GSK1016790A in activating the TRPV4 current [25]. 4αPDD can also act through other pathways [26,27]. Although it is necessary to have further investigation, the present results suggest that TRPV4 is involved in the upregulation of *β-casein* and TJ protein-encoding transcript levels upon mild heat shock at 39 °C.

Our present study indicates that TRPV4 activation with GSK1016790A increased the *Xbp1s* transcript level. TRPV4 activation leads to Ca^2+^ influx [28,29]. The mammalian UPR is a highly preserved signaling cascade that is commenced during endoplasmic reticulum (ER) stress conditions. The ER is an organelle that is responsible for the storage of Ca^2+^. It is reported that ER Ca^2+^ depletion activates XBP1 [30]. Activation of TRPV4 channels after intracerebral hemorrhage leads to the destruction of Ca^2+^ homeostasis, which in turn caused UPR [31]. Though further investigation is necessary, it is speculated that TRPV4-mediated Ca^2+^ signaling regulates *Xbp1s* expression. In addition, the increased mRNA expressions of *β-casein*, *Zo-1*, *Ocln*, and *Cldn3* by mild heat treatment was inhibited by Xbp1 knockdown. The results suggest that XBP1 regulates the expression of the TJ protein-encoding genes. This result is consistent with previous reports that the IRE1-XBP1 pathway regulates retinal pigment epithelium TJs [32]. Because XBP1 is a transcription factor, it will be necessary to clarify whether it directly regulates the expression of these genes.

We found that heat treatment at 41 °C, but not 39 °C, significantly increased the transcript levels of *Atf4* and *Chop*. Moreover, only heat treatment at 41 °C significantly decreased cell viability. When excessive ER stress is initiated or continued for a long period of time, cells undergo apoptosis. The UPR pathway protects cells from apoptotic death in response to stress stimulus, but can also lead to apoptosis when it is difficult to adapt to the stress [33]. The UPR is facilitated by three ER receptors: PERK, IRE1, and ATF6. Among these proteins, PERK is essential for the induction expression of the proapoptotic transcriptional factor CHOP under ER stress conditions and is dominant over the ATF6 and IRE1/XBP-1 signaling pathways [34]. It is thought that these changes are associated with a decrease in cell viability. Temperatures greater than 41 °C to 42 °C can lead to cell death from apoptosis within a few hours [35]. Conversely, it is well known that mild heat stress (39–40 °C) can positively regulate cell proliferation and differentiation [6]. TRP channels cover the range of temperature sensed. Therefore, TRPV4 is activated at 39 °C, but different TRPs may be activated at 41 °C.

**Figure 6:**
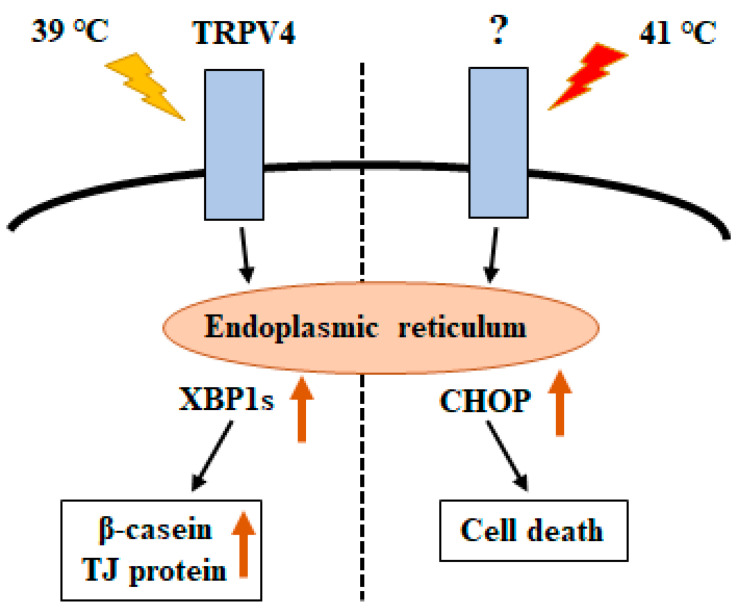
Schematic model showing unfolded protein response (UPR)-mediated crosstalk between temperature and cellular response.

Although further mechanistic examination is necessary, it is speculated that the difference in UPR gene expression upon stimulation at 39 °C vs. 41 °C controls cell survival vs. cell death (Figure 6).

It is well known that metabolic heat is produced during milk production in the lactating mammary gland [36]. This exposes mammary epithelial cells to temperatures above body temperature. Therefore, mild heat stress may have the effect of enhancing or maintaining milk production in vivo. On the other hand, severe heat stress (41 °C) significantly decreased cell viability with elevating the *Chop* mRNA levels. It has been reported that milk production decreases with a decrease in the number of cells in the mammary gland tissue [37]. This decrease in the number of cells is because the rate of cell death by apoptosis is higher than the rate of proliferation [38,39]. We demonstrated that milk production is lower in individuals with higher CHOP expression in mammary tissue of dairy cows [40]. Although detailed examination using dairy cows is necessary in the future, one of the factors causing a decrease in milk production by heat stress could be mammary epithelial cell death arising from ER stress.

Mammary alveoli are formed during mid-gestation to late gestation [1]. The stromal extracellular matrix (ECM) plays a significant role in mammary epithelial behavior as well as in branching morphogenesis through its mechanical efforts [41]. For example, filamin A (an integrin-linked scaffold protein) drives a mechanism controlling the physical properties of ECM, thereby regulating the morphogenesis [42]. TRPV4 is activated not only by temperature but also by mechanical forces [43]. The previous study demonstrated that mechanical forces can upregulate TRPV4 expression and also physically impair ECM, thereby regulating the capillary cell reorientation [44]. Moreover, a previous analysis of claudin mRNA in the mammary gland at different stages of maturation indicated that *Cldn3* mRNA had the highest expression level at mid-pregnancy and fell about 10-fold at the onset of lactation [45]. Therefore, the expression of both *Trpv4* and *Cldn3* mRNA increased until Day 15 of pregnancy, and decreased similarly afterward. A recent study suggested that TRPV4 regulated TJs and affected the corneal epithelial cell differentiation [23]. Although further research is necessary, TRPV4 activation, which increased the strength of TJs, may be involved in normal mammary gland development.

## 5. Conclusions

In conclusion, the results indicate that mild heat stress induces the transcriptional level of *Xbp1s* via TRPV4 activity, which enhances the expression of the *β-casein* and TJ protein-encoding genes. On the other hand, severe heat stress induces the transcriptional level of *Chop*, which reduces the cell viability. It is speculated that the difference in UPR gene expression upon stimulation at 39 °C vs. 41 °C controls cell survival vs. cell death. Moreover, our data suggest that *Trpv4* expression increased during gestation but decreased during lactation. These findings provide new insights into the possible function of TRPV4 in mammary gland development.

## Figures and Tables

**Table 1:** Sequences of primers used for real-time PCR amplification.

Gene	Primers (5′ to 3′)
*Zo-1*	Forward GGGAGGGTCAAATGAAGACA Reverse GGCATTCCTGCTGGTTACAT
*Cldn3*	Forward AGCCAGTCTCCAAAGCCACA Reverse CTGGGAATCAACTGCCCTTC
*Ocln*	Forward CGGACCCTGACCACTATGAAA Reverse CCTGCAGACCTGCATCAAAA
*Trpv4*	Forward ATGGCAGATCCTGGTGATGG Reverse GGAACTTCATACGCAGGTTTGG
*β-casein*	Forward GATGCCCCTCCTTAACTCTGAA Reverse TTAGCAAGACTGGCAAGGCTG
*Xbp1s*	Forward TGAGAACCAGGAGTTAAGAACACGC Reverse CCTGCACCTGCTGCGGAC
*Ire1α*	Forward ACGAAGGCCTGACGAAACTT Reverse ATCTGAACTTCGGCATGGGG
*Atf4*	Forward GAGCTTCCTGAACAGCGAAGTG Reverse TGGCCACCTCCAGATAGTCATC
*Chop*	Forward CCTAGCTTGGCTGACAGAGG Reverse CTGCTCCTTCTCCTTCATGC
*Atf6α*	Forward CTTCCTCCAGTTGCTCCATC Reverse CAACTCCTCAGGAACGTGCT
*Grp78*	Forward GAAAGGATGGTTAATGATGCTGAG Reverse GTCTTCAATGTCCGCATCCTG
*Gapdh*	Forward TTGTGATGGGTGTGAACCACGAG Reverse CATGAGCCCTTCCACAATGCCAA
